# Purification and Characterization of Botulinum Neurotoxin FA from a Genetically Modified *Clostridium botulinum* Strain

**DOI:** 10.1128/mSphere.00100-15

**Published:** 2016-02-24

**Authors:** Sabine Pellett, William H. Tepp, Marite Bradshaw, Suzanne R. Kalb, Janet K. Dykes, Guangyun Lin, Erin M. Nawrocki, Christina L. Pier, John R. Barr, Susan E. Maslanka, Eric A. Johnson

**Affiliations:** a Department of Bacteriology, University of Wisconsin−Madison, Madison, Wisconsin, USA; b National Center for Emerging and Zoonotic Infectious Diseases, Centers for Disease Control and Prevention, Atlanta, Georgia, USA; c National Center for Environmental Health, Centers for Disease Control and Prevention, Atlanta, Georgia, USA; Swiss Federal Institute of Technology Lausanne

**Keywords:** BoNT/FA, *Clostridium botulinum*, botulinum neurotoxin, chimeric toxin

## Abstract

Botulinum neurotoxins (BoNTs), produced by neurotoxigenic clostridial species, are the cause of the severe disease botulism in humans and animals. Early research on BoNTs has led to their classification into seven serotypes (serotypes A to G) based upon the selective neutralization of their toxicity in mice by homologous antibodies. Recently, a report of a potential eighth serotype of BoNT, designated “type H,” has been controversial. This novel BoNT was produced together with BoNT/B2 in a dual-toxin-producing *Clostridium botulinum* strain. The data used to designate this novel toxin as a new serotype were derived from culture supernatant containing both BoNT/B2 and novel toxin and from sequence information, although data from two independent laboratories indicated neutralization by antibodies raised against BoNT/A1, and classification as BoNT/FA was proposed. The sequence data indicate a chimeric structure consisting of a BoNT/A1 receptor binding domain, a BoNT/F5 light-chain domain, and a novel translocation domain most closely related to BoNT/F1. Here, we describe characterization of this toxin purified from the native strain in which expression of the second BoNT (BoNT/B) has been eliminated. Mass spectrometry analysis indicated that the toxin preparation contained only BoNT/FA and confirmed catalytic activity analogous to that of BoNT/F5. The *in vivo* mouse bioassay indicated a specific activity of this toxin of 3.8 × 10^7^ mouse 50% lethal dose (mLD_50_) units/mg, whereas activity in cultured human neurons was very high (50% effective concentration [EC_50_] = 0.02 mLD_50_/well). Neutralization assays in cells and mice both indicated full neutralization by various antibodies raised against BoNT/A1, although at 16- to 20-fold-lower efficiency than for BoNT/A1.

**IMPORTANCE** Botulinum neurotoxins (BoNTs), produced by anaerobic bacteria, are the cause of the potentially deadly, neuroparalytic disease botulism. BoNTs have been classified into seven serotypes, serotypes A to G, based upon their selective neutralization by homologous antiserum, which is relevant for clinical and diagnostic purposes. Even though supportive care dramatically reduces the death rate of botulism, the only pharmaceutical intervention to reduce symptom severity and recovery time is early administration of antitoxin (antiserum raised against BoNTs). A recent report of a novel BoNT serotype, serotype H, raised concern of a “treatment-resistant” and highly potent toxin. However, the toxin’s chimeric structure and characteristics indicate a chimeric BoNT/FA. Here we describe the first characterization of this novel toxin in purified form. BoNT/FA was neutralized by available antitoxins, supporting classification as BoNT/FA. BoNT/FA required proteolytic activation to achieve full toxicity and had relatively low potency in mice compared to BoNT/A1 but surprisingly high activity in cultured neurons.

## Abstract (precis)

Botulinum neurotoxins (BoNTs), produced by anaerobic bacteria, are the cause of the potentially deadly, neuroparalytic disease botulism. BoNTs have been classified into seven serotypes, serotypes A to G, based upon their selective neutralization by homologous antiserum, which is relevant for clinical and diagnostic purposes. Even though supportive care dramatically reduces the death rate of botulism, the only pharmaceutical intervention to reduce symptom severity and recovery time is early administration of antitoxin (antiserum raised against BoNTs). A recent report of a novel BoNT serotype, serotype H, raised concern of a “treatment-resistant” and highly potent toxin. However, the toxin’s chimeric structure and characteristics indicate a chimeric BoNT/FA. Here we describe the first characterization of this novel toxin in purified form. BoNT/FA was neutralized by available antitoxins, supporting classification as BoNT/FA. BoNT/FA required proteolytic activation to achieve full toxicity and had relatively low potency in mice compared to BoNT/A1 but surprisingly high activity in cultured neurons.

## INTRODUCTION

Botulinum neurotoxins (BoNTs) are potent agents of paralysis and the cause of botulism, a rare but potentially fatal disease in humans and animals (1, 2). Even though botulism is a rare disease in developed countries, with only about 150 cases per year in the United States, it remains a significant concern for several reasons. First, botulism can be severe and of long duration, resulting in a high degree of human suffering and high associated health care costs and with a mortality rate of about 5% in developed countries. Second, *Clostridium botulinum* is widespread in nature and forms spores that can survive many standard antibacterial treatments, such that botulism remains a perennial concern in the food industry. Third, there is no cure for botulism, and treatment is restricted to supportive care, including mechanical ventilation in severe cases, and administration of antitoxin, which is most effective if given within the first 72 h after ingestion of neurotoxins in contaminated food. The antitoxin contains equine-derived polyclonal antibodies to the seven known BoNT serotypes (3). Administration of antitoxin results in neutralization of circulating toxin but is ineffective against toxin that has already entered neuronal cells. After neuronal cell entry, which occurs mainly during the first 3 days after the toxin enters the body, BoNTs can have an extraordinarily long duration of action inside neuronal cells, leading to the prolonged flaccid paralysis characteristic of botulism (2). During this time, intensive supportive care is required, and the antitoxin is ineffective. In infant botulism (and in very rare cases in adults), colonization of *C. botulinum* occurs in the immature or immunocompromised intestine, leading to continuous toxin production (4). Because toxin continues to be produced by *C. botulinum* in the intestine, neutralizing antibodies in the circulation systems of these patients can continue to reduce the severity and duration of symptoms, and a human IgG (BabyBIG) for treatment of these cases has been developed to avoid the side effects associated with equine antitoxin (5).

Due to the high potency of BoNTs, the lack of a cure, the potentially high mortality rate in the absence of treatment, and the high cost and limited availability of the required intensive supportive care, these toxins are considered a potential bioterrorism weapon and remain of high national and international significance (6, 7). BoNTs are produced by a diverse group of anaerobic bacteria designated *Clostridium botulinum* and a few strains of otherwise atoxic *Clostridium* species, including *C. baratii*, *C. butyricum*, and *C. sporogenes* (8, 9). BoNTs have been classified into seven serotypes, designated BoNT/A through BoNT/G, on the basis of their specific neutralization by antiserum raised against the homologous serotype (10, 11), and are further subdivided into subtypes/variants (12, 13). Most strains of *C. botulinum* produce only a single serotype of BoNT. However, a number of strains are known to produce two or even three different BoNTs, with one of the toxins usually dominant as determined by activity in mice (10, 12–21).

One such bivalent strain was reported in 2014 from an infant botulism case. This strain, designated *C. botulinum* strain IBCA10-7060, was isolated and cultured, and neutralization experiments and immunological studies of culture supernatant indicated the presence of BoNT/B and an additional unknown BoNT that was only weakly neutralized by the antibodies against currently known serotypes that were used in the study. This led the authors to suggest designation as a novel serotype, “type H” (22, 23). Following publication of this study, strain IBCA10-7060 was sent to the Centers for Disease Control and Prevention (CDC), where it was subcultured, and the subculture received the designation *C. botulinum* CDC69016. Further genetic analyses of strain CDC69016 indicated that this toxin has a chimeric structure consisting of a light chain (LC) similar to the LC of BoNT/F5, a receptor binding domain similar to the receptor binding domain BoNT/A1, and a complex translocation domain similar to the complex translocation domain of BoNT/F1 (23–25). Detailed studies of CDC69016 culture supernatants indicated that toxicity was fully neutralized by a combination of anti-BoNT/B and anti-BoNT/A antisera (26). Considering these data and resemblance of this toxin to the chimeric mosaic toxins found within serotypes C and D (27, 28), a designation of this toxin as BoNT/FA may be more descriptive than the original designation as a new serotype H.

However, all of these studies had been undertaken using crude culture supernatants containing two toxins or by attempted neutralization of type B in the crude culture, which can complicate the efficacy and interpretation of neutralization. The isolation of BoNT/FA toxin presented a challenge due to the production of BoNT/B2 by the same strain. Standard biochemical separation of two different BoNTs from the same strain is generally difficult, and even if the toxins can be separated, the possibility that there are small quantities of the second BoNT in the preparations cannot be excluded. With the increasing availability of DNA sequences of the BoNT genes, one approach for selective production of one of the BoNTs is by recombinant expression in native hosts (29, 30) or heterologous hosts (reviewed in reference 31). Posttranslational modifications of BoNT that occur in *C. botulinum* likely do not occur by the same mechanism in heterologous hosts. Therefore, in our view, it is preferable to use a native host for the production of a novel BoNT for subsequent characterization. This requires either expression of a recombinant BoNT from an expression plasmid in an atoxic *C. botulinum* expression host or selective inactivation of the second BoNT gene in a dual-toxin-expressing strain. Selective inactivation of the BoNT genes in *C. botulinum* by ClosTron technology has been described previously (30, 32, 33). Here we describe an approach in which the gene encoding BoNT/B2 was selectively inactivated by ClosTron technology (34, 35) in strain CDC69016, enabling the singular expression of the BoNT/FA toxin for purification and characterization. This enabled purification of BoNT/FA, allowing for more accurate determination of its potency and immunological characterization in mice and in neuronal cells.

## RESULTS

### Expression of the BoNT/B2 in strain CDC69016 can be eliminated while maintaining expression of BoNT/FA.

**FIG 1:**
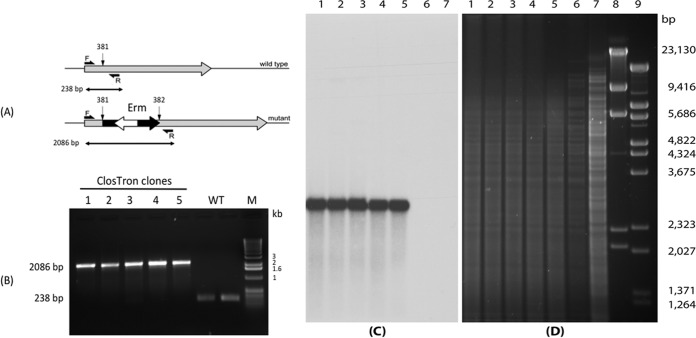
Genetic disruption of the *bont*/B2 gene in strain CDC69016. (A) Schematic presentation of the wild-type (top) and mutated (bottom) botulinum neurotoxin B2 gene. The group II intron is shown as a black wide arrow inserted on the sense strand of the toxin gene (gray arrow) between nucleotides 381 and 382 as indicated by the vertical black arrows. The white arrow inside the intron element in the opposite orientation to the intron and toxin gene is a retrotransposition-activated erythromycin (RAM-Erm) resistance gene. The locations of the forward (F) and reverse (R) PCR primers are shown with horizontal arrows on either side of the intron insertion site. The expected size of the PCR products for the wild-type (WT) strain is 238 bp, and it is 2,086 bp for the inactivated BoNT/B2 gene. (B) PCR products of five putative mutant clones (clones 1 to 5) and two samples of the wild-type (WT) strain. The positions of molecular size markers (M) (Track-it DNA ladder [Life Technologies]) in base pairs are shown to the right of the gel. (C and D) Southern hybridization with the intron probe (erythromycin gene) (C) and ethidium bromide-stained 1% agarose gel of genomic DNA digested with restriction enzyme HindIII (D). Lanes 1 to 5, five individual putative BoNT/B2 mutant clones; lane 6, wild-type CDC69016 strain; lane 7, *C. botulinum* strain CDC-A3 as a negative control; lanes 8 and 9, DNA markers (NEB, Ipswich, MA). The sizes of the DNA markers (in base pairs) are indicated to the right of the gel.

To facilitate purification and characterization of BoNT/FA from the dual-toxin-producing strain CDC69016, production of BoNT/B2 was genetically eliminated in this strain by inactivation of the *bont/*B2 gene using the ClosTron mutagenesis system (34, 35). The inactivation of the *bont*/B2 gene was confirmed by PCR, sequencing, and Southern hybridization analysis (Fig. 1), and the resultant strain was designated CDC69016/B2tox^−^.

**FIG 2:**
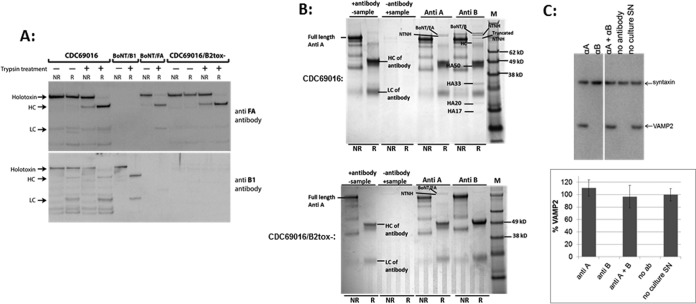
Elimination of BoNT/B2 production in strain CDC69016/B2tox^−^ while BoNT/FA production is maintained. (A) Western blot analysis of reduced (R) and nonreduced (NR) culture supernatants of *C. botulinum* strains CDC69016 and CDC69016/B2tox^−^ after growth for 5 days at 37°C. BoNT/FA and BoNT/B2 were detected with BoNT/FA-specific antibody (anti FA antibody) and BoNT/B1-specific antibodies (anti B1 antibody), respectively. The 150-kDa BoNT/B and BoNT/FA bands are indicated. (B) Immunoprecipitation analysis of the toxin complexes produced in *C. botulinum* strains CDC69016 and CDC69016/B2tox^−^. The BoNT/FA and BoNT/B2 toxin complexes were immunoprecipitated from culture supernatants using polyclonal antibodies raised against BoNT/A1 or BoNT/B1. The proteins recovered were separated by SDS-PAGE and visualized by staining with Coomassie blue. Abbreviations: NR, nonreduced samples; R, samples reduced with 100 mM DTT; LC, light chain; HC, heavy chain; kD, kilodaltons. The nontoxigenic components of the BoNT complex were nontoxic nonhemagglutinin (NTNH) and HA50, HA33, HA20, and HA17 hemagglutinin complex proteins. For negative controls, beads with BoNT/A1 antibody only and beads with culture supernatant only were used as indicated. (C) Cell-based neutralization assay. A trypsinized culture supernatant of *C. botulinum* strain CDC69016/B2tox^−^ was examined for the presence of BoNT/FA and BoNT/B2 toxin activity by antibody neutralization with anti-BoNT/A1 or anti-BoNT/B1 antibodies in hiPSC-derived neurons. Cell lysates were analyzed by Western blotting using monoclonal antibodies specific to syntaxin and VAMP2 (Synaptic Systems). The bands on the Western blot were quantified by densitometry using TotalLab Quant software (Fotodyne), and the percentage of VAMP2 in relation to syntaxin (as the loading control) was determined. The graph shows the average plus standard deviation (error bar) values of three replicates. αA, anti-type A antibody; SN, supernatant; ab, antibody.

Western blot analysis of strains CDC69016 and CDC69016/B2tox^−^ grown for 5 days at 37°C with anti-BoNT/FA and anti-BoNT/B1 antibodies resulted in 150-kDa BoNT/B and BoNT/FA bands in the wild-type strain CDC69016 and in only a 150-kDa BoNT/FA band in the CDC69016/B2tox^−^ mutant (Fig. 2A). This indicates that BoNT/B2 is not produced in the CDC69016/B2tox^−^ strain. Furthermore, even though all samples were reduced prior to separation by sodium dodecyl sulfate-polyacrylamide gel electrophoresis (SDS-PAGE), only a small percentage of the BoNT/B2 protein appeared as bands specific for heavy chain (HC) (100 kDa) or light chain (LC) (50 kDa) in addition to the more-prominent 150-kDa holotoxin band. For BoNT/FA, only a 150-kDa band and no HC or LC bands were observed. This indicates that although these cultures are proteolytic (22, 25), only BoNT/B2 toxin is partially endogenously converted to the dichain form in the wild-type CDC69016 strain and BoNT/FA was not converted to the dichain form in either the wild-type strain or the CDC69016/B2tox^−^ strain.

The absence of BoNT/B2 expression in strain CDC69016/B2tox^−^ was further demonstrated by immunoprecipitation analyses of the culture supernatants from both the wild-type and mutant strains using antibodies raised against BoNT/A1 for detection of the BoNT/FA toxin complex and antibodies raised against BoNT/B1 for detection of the BoNT/B2 complex. SDS-PAGE gel analysis revealed that both BoNT/FA and BoNT/B2 and their respective complex proteins (nontoxic nonhemagglutinin protein [NTNH] for BoNT/FA and NTNH, HA50, HA33, HA20, and HA17 for BoNT/B2) were immunoprecipitated from the wild-type strain CDC69016 (Fig. 2B). For BoNT/FA, its complex protein NTNH, but not BoNT/B complex proteins (NTNH and hemagglutinins [HAs]) were immunoprecipitated from the BoNT/B2tox^−^ mutant (Fig. 1B), indicating that the BoNT/FA does not associate with the complex proteins from the inactivated BoNT/B. These data confirmed that the BoNT/B2tox^−^ strain produces the BoNT/FA toxin but not the BoNT/B2 toxin at levels detectable by immunoprecipitation.

**FIG 3:**
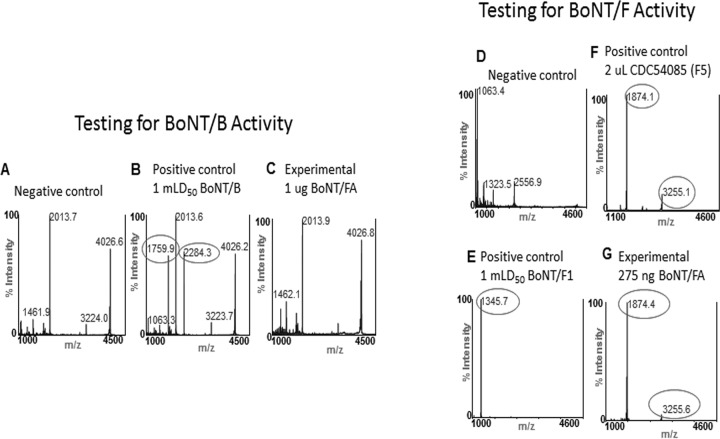
MS analysis demonstrating purity of the isolated BoNT/FA and *in vitro* cleavage properties. The purified BoNT/FA was analyzed by endopeptidase mass spectrometry (Endopep-MS) for its BoNT/B-like (left) and BoNT/F-like (right) endopeptidase activity using specific VAMP fragments. No cleavage was seen for the BoNT/B peptide, SubB (*m/z* 4024.6). BoNT/B cleavage results with peptides at *m/z* 1760.0 and *m/z* 2283.6. Only BoNT/F-like activity was detected, and the substrate, SubF (*m/z* 5111.1), was cleaved between L-28 and E-29 to yield cleavage products at *m/z* 3255.1 corresponding to the N-terminal cleavage product and *m*/*z* 1874.0 for the C-terminal cleavage product. These are the same amino acid residues that BoNT/F5 cleaves, but not at the residues cleaved by BoNT/F1 (Q-32 and K-33, yielding cleavage products at *a* 1345.6 and 3781.5).

Additionally, the BoNT/FA material was tested for BoNT/B enzymatic activity. Figure 3 shows the mass spectra of the reactions of BoNT/B and BoNT/FA with peptide substrate SubB. The peaks at *m/z* 4027 and 2014 in the negative control in Fig. 3A correspond to singly charged and doubly charged intact SubB. These peaks are also in the reaction of 9 ng of BoNT/B with SubB in Fig. 3B; however, that spectrum also includes two new peaks at *m/z* 1760 and 2284. The peak at *m/z* 1760 corresponds to LSELDDRADALQAGASQ, or the N-terminal cleavage product of SubB by BoNT/B; the peak at *m/z* 2283 is FESSAAKLKRKYWWKNLK, the C-terminal cleavage product of SubB. The BoNT/B in the culture supernatant containing BoNT/FA was previously shown to produce the same cleavage products upon incubation with SubB (24). Figure 3C shows the mass spectrum of the reaction of 1 µg of the BoNT/FA material, several orders of magnitude more toxin than used in the positive-control reaction, and the BoNT/B cleavage products are not seen in this reaction, indicating the absence of enzymatically active BoNT/B in this purified BoNT/FA material.

The amino acid sequence of the purified BoNT/FA was also examined by digesting 1 µg of the material with trypsin followed by liquid chromatography coupled to tandem mass spectrometry (LC-MS/MS) analysis of the resultant tryptic peptides. MS/MS sequence data were obtained for 90.5% of the protein (data not shown), and no evidence was obtained to show the existence of changes in this toxin’s amino acid sequence or to show the existence of another toxin such as BoNT/B.

Finally, trypsinized culture supernatant from the CDC69016/B2tox^−^ mutant strain was analyzed for BoNT/FA and BoNT/B activity by a neuronal cell-based neutralization assay (Fig. 2C). Exposure of human induced pluripotent stem cell (hiPSC) neurons to the BoNT/B2tox^−^ mutant culture supernatant resulted in disappearance of the VAMP2 band (Fig. 2C). VAMP2 is the target substrate for both BoNT/FA and BoNT/B2, and therefore, specific anti-A and anti-B antibodies were used in a neutralization assay to determine toxin-specific cleavage. VAMP2 cleavage was completely prevented by preincubating the culture supernatant with only anti-BoNT/A1 antibodies but not with anti-BoNT/B1 antibodies (Fig. 2C). This indicates that all neurotoxin activity detected in the CDC69016/B2tox^−^ mutant is due to the BoNT/FA toxin, which is neutralized by polyclonal anti-BoNT/A1 antibody (26). These results further confirmed the absence of BoNT/B2 production in the CDC69016/B2tox^−^ mutant strain.

### Trypsinization increases BoNT/FA activity.

**FIG 4:**
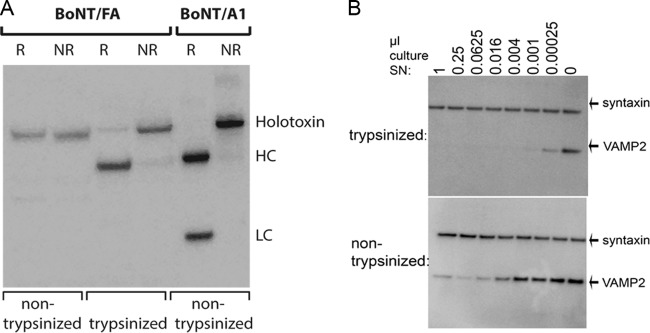
Analysis of BoNT/FA activity with and without trypsin treatment. The culture supernatant of the BoNT/B2tox^−^ mutant strain was either treated with trypsin for 60 min at 37°C, followed by trypsin inactivation by the addition of soybean trypsin inhibitor, or not treated with trypsin. (A) Western blot showing reduced (R) and nonreduced (NR) samples of trypsin-treated and nontreated BoNT/FA from concentrated culture supernatant detected with antibodies raised against BoNT/A1. Purified BoNT/A1 (reduced and nonreduced) was loaded on a gel as a positive control. HC, heavy chain; LC, light chain. (B) Western blot showing activity of trypsin-treated versus nontreated BoNT/FA in cultured neurons. HiPSC-derived neurons were incubated with the indicated amounts of either trypsin-treated or nontreated culture supernatant (SN) for 48 h, and VAMP2 cleavage was assessed by Western blotting comparing the VAMP2 signal to the syntaxin signal (loading control).

In order to determine toxin expression by the CDC69016/B2tox^−^ strain, the BoNT activity of culture supernatant after growth for 6 days in toxin production medium (TPM) (components of TPM given in Materials and Methods) at 37°C was determined by mouse bioassay. The toxicities of two independent batches of untrypsinized culture supernatants were both ~280 mouse 50% lethal dose (mLD_50_) per ml. BoNT toxins usually require posttranslational proteolytic cleavage (“nicking”) between the HCs and LCs of the toxins to achieve full activity. This step usually occurs naturally in group I proteolytic *C. botulinum* strains. Even though a previous genomic analysis of strain CDC69016 has indicated that this strain is most closely related to group I proteolytic *C. botulinum* (23, 25), Western blot analysis of culture supernatants from this strain has indicated that both BoNTs in this strain are not proteolytically processed into the HC-LC dimer (Fig. 2A). Therefore, the effect of trypsin treatment on the toxicity of culture supernatant from strain CDC69016/B2tox^−^ was assessed to determine whether “nicking” of BoNT/FA by an exogenous enzyme affects its toxicity. The supernatants from the two independent batches of strain CDC69016/B2tox^−^ grown for 6 days in TPM at 37°C were treated with trypsin, resulting in “nicking” of the toxin to a dichain form (Fig. 4A). The toxin activity of trypsin-treated and nontreated supernatant was determined by mouse bioassay and by cell-based assay. In both assays, the toxin activity was increased dramatically after trypsin treatment. The mouse bioassay resulted in ~11,300 mLD_50_/ml for batch 1 and ~10,080 mLD_50_/ml for batch 2 for trypsinized culture supernatant versus ~280 mLD_50_/ml for nontrypsinized culture supernatant, giving an ~38-fold increase. The values of 11,300 and 10,080 mLD_50_/ml are similar to the estimate of 11,200 mLD_50_/ml for BoNT/FA toxicity determined in trypsin-treated culture supernatant from the wild-type *C. botulinum* strain CDC69016 culture in a previous study (26).

The cell-based activity assay using hiPSC-derived neurons resulted in dose-dependent VAMP2 cleavage that was complete at quantities greater than 0.004 µl of culture supernatant per well for trypsin-treated culture supernatant (Fig. 4B). However, VAMP2 cleavage was significantly less efficient with nontrypsinized culture supernatant. A volume of 0.016 µl of nontrypsinized culture supernatant per well was required to detect VAMP2 cleavage, and complete cleavage was not reached even with as much as 1 µl of nontrypsinized culture supernatant per well. This indicates that in hiPSC-derived neurons, similar to the data obtained in mice, the trypsinized BoNT/FA toxin is greater than 20-fold more active than nontrypsinized toxin.

### BoNT/FA can be isolated from the CDC69016/B2tox^−^ strain and cleaves VAMP2 at the same cleavage site as BoNT/F5.

**FIG 5:**
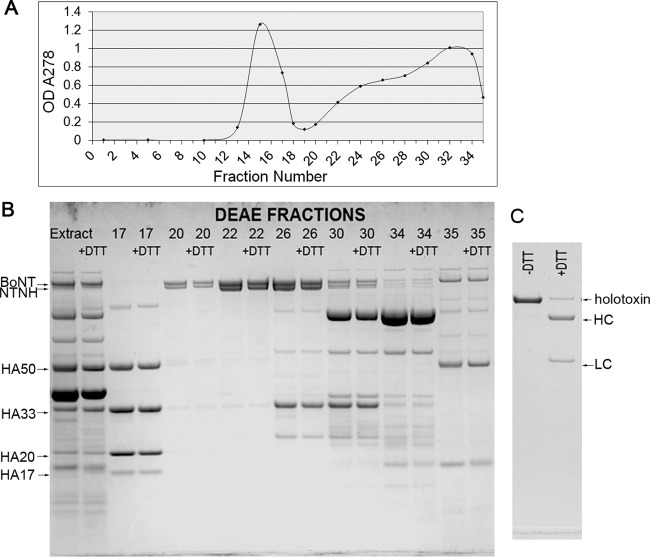
Purification of BoNT/FA from strain CDC69016/B2tox^−^. (A) Chromatogram showing the OD_278_ or *A*_278_ for the fractions collected from the DEAE-Sephadex A-50 column at pH 5.5 to separate the FA complex from other proteins present in the crude toxin extract from strain CDC69016/B2tox^−^. (B) SDS-polyacrylamide gel showing the protein bands of selected fractions from the same column under reduced (+DTT) and nonreduced (−DTT) conditions. Most of the BoNT/FA complex consisting of BoNT/FA and NTNH was eluted in the ascending portion of the second peak (fractions 20 to 27), while the NTNH-HA complexing proteins normally associated with the BoNT/B2 were eluted in the first peak (fractions 13 to 18). Fractions 28 to 35 show progressively less FA complex along with increasing amounts of nontarget proteins. (C) SDS-polyacrylamide gel showing the purified BoNT/FA under reduced (+DTT) and nonreduced (−DTT) conditions.

The toxin activity in culture supernatants of strain CDC69016/B2tox^−^ indicated that sufficient toxin was produced by this strain for standard biochemical purification used for other botulinum neurotoxins. The activity of a trypsin-treated toxin extract prepared from *C. botulinum* strain CDC69016/B2tox^−^ was determined by the mouse bioassay to contain about 2.3 × 10^8^ mouse LD_50_/ml. About 52 ml of this extract was used for toxin purification. Chromatography of the crude and trypsin-treated toxin extracts on a DEAE-Sephadex A-50 column at pH 5.5 resulted in separation of the BoNT/FA complex consisting of BoNT/FA and NTNH as well as the NTNH-HA complex proteins normally associated with the BoNT/B2 of the native culture (Fig. 5). Most of the BoNT/FA complex was eluted in the ascending portion of the second peak from the DEAE-Sephadex column at pH 5.5, while the NTNH-HA complex proteins normally associated with BoNT/B2 were eluted in the first peak. Fractions approaching the apex and descending portion of the second peak contained progressively less FA complex along with increasing amounts of nontarget proteins and were thus excluded from the FA complex pool. SDS-PAGE analysis of the BoNT/FA complex recovered from the first DEAE column showed the ~140-kDa NTNH band and an ~150-kDa toxin band even after reduction, indicating that the toxin was in the single-chain form and not the dichain form (Fig. 5B). The complex was again treated with trypsin to convert the single-chain toxin to its fully active dichain form. A second chromatography step, a DEAE-Sephadex column at pH 7.6, was used to separate the ~150-kDa BoNT/FA toxin from the NTNH. The FA toxin eluted from the column in the first peak after application of a linear sodium chloride gradient (0 to 350 mM in 0.01 M sodium phosphate buffer [pH 7.6]). The purified and “nicked” 150-kDa toxin was demonstrated by SDS-PAGE under both unreduced and reduced conditions (Fig. 5C), and protein concentration was estimated by *A*_278_ using the extinction coefficient previously determined for BoNT/A1 (36). Isolation of BoNT/FA was achieved at greater than 95% purity as determined by densitometry (Fig. 5C). The BoNT/FA obtained in the final step consisted of ~85 to 90% dichain toxin and ~10 to 15% single-chain toxin. An estimated 1.05 mg of pure BoNT/FA was recovered from approximately 6 liters of starting culture.

BoNT/F1 is known to cleave VAMP2 and shorter peptides based on the sequence of VAMP2 between ^58^Q and ^59^K (37). Figure 3D to G show the mass spectra of the reactions of BoNT/F1, BoNT/F5, and BoNT/FA with a shortened form of VAMP2, SubF. The peak at *m/z* 2556.9 in the negative control of Fig. 3D is doubly charged intact SubF. Upon reaction with BoNT/F1, SubF is cleaved to yield two cleavage products corresponding to cleavage between ^58^Q and ^59^K of VAMP2: the N-terminal cleavage product at *m/z* 3782.9 (not shown) corresponding to TSNRRLQQTQAQVDEVVDIMRVNVDKVLERDQ and the C-terminal cleavage product at *m/z* 1345.7 corresponding to KLSELDDRADAL in Fig. 3E. The N-terminal cleavage product produced by cleavage of SubF by BoNT/F1 is a long peptide and historically is rarely seen with this type of matrix-assisted laser desorption ionization−time of flight mass spectrometry (MALDI-TOF MS) analysis (24); therefore, the C-terminal cleavage product at *m/z* 1345.7 is indicative of the enzymatic activity of BoNT/F1 upon SubF.

Both BoNT/F5 and BoNT/FA have been reported to cleave VAMP2 and shorter peptides based on the sequence of VAMP2 between ^54^L and ^55^E (24), four amino acids upstream from the cleavage site by other BoNT/F (37). Figure 3F shows the mass spectrum of the reaction of BoNT/F5 with SubF. The peak at *m/z* 3255.1 corresponds to TSNRRLQQTQAQVDEVVDIMRVNVDKVL, the N-terminal cleavage product of SubF, and the peak at *m/z* 1874.1 corresponds to the C-terminal cleavage product, ERDQKLSELDDRADAL. These same peaks are present in Fig. 3G, which shows the mass spectrum of the reaction of BoNT/FA with SubF, indicating that this purified BoNT/FA material is enzymatically active and has the same enzymatic function known for BoNT/FA purified from culture supernatant.

### BoNT/FA has a lower specific activity in mice than BoNT/A1 but has a higher activity in cultured human neurons than BoNT/A1.

The specific toxicity of the purified BoNT/FA toxin was determined by mouse bioassay. While in a typical mouse bioassay with BoNT/A1, most mice die within the first 24 to 48 h and rarely die after 72 h, mice injected with BoNT/FA appeared healthy within the first 24 to 48 h and up to 6 days were required to complete the mouse bioassay (Table 1). The specific activity of the 150-kDa BoNT/FA was determined to be ~3.8 × 10^7^ LD_50_/mg, which is approximately five times lower than that of BoNT/A1, and indicates an about 17% yield of toxin purified from the crude extract.

**TABLE 1:** Mouse bioassay to determine the specific activity of BoNT/FA

Amt (pg) of toxin/mouse	No. of dead mice/total no. of mice injected with toxin on:					
	Day 1	Day 2	Day 3	Day 4	Day 5	Day 6
90	0/4	4/4				
60	0/4	2/4	4/4			
50	0/4	2/4	4/4			
40	0/4	0/4	4/4			
30	0/4	0/4	0/4	0/4	2/4	3/4
20	0/4	0/4	0/4	0/4	0/4	0/4

**FIG 6:**
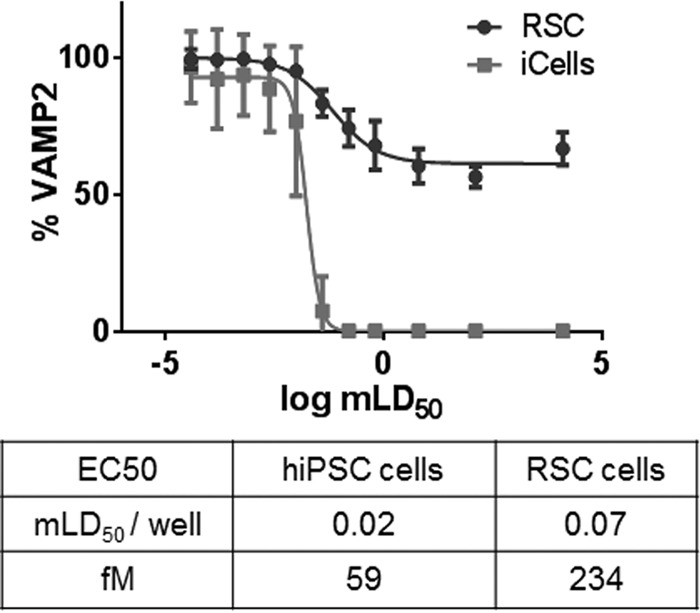
Activity of BoNT/FA in cultured neurons shown by quantitative depiction of VAMP2 cleavage in cultures of primary rat spinal cord (RSC) cells and human induced pluripotent stem cells (hiPSC)-derived neurons. The cells were exposed to serial dilutions of the purified BoNT/FA toxin for 48 h, and cell lysates were analyzed by Western blotting using monoclonal antibodies specific to syntaxin and VAMP2 (Synaptic Systems). The bands on the Western blot were quantified by densitometry using TotalLab Quant software (Fotodyne), and the percentage of VAMP2 in relation to syntaxin (as the loading control) was determined. The graph was created with Prism6 software using a nonlinear regression analysis and show the average ± standard deviation (error bar) values of three replicates. The EC_50_ values estimated from the regression analysis are shown below the graph.

The activity of the purified BoNT/FA toxin was further examined in two different cell models, cultured primary rat spinal cord (RSC) cells and hiPSC-derived neurons. Western blot analysis of cell lysates of the exposed cells showed that with increasing concentrations of BoNT/FA, the VAMP2 in the human cell model dropped to levels below the detectable range, whereas the VAMP2 in the RSC cell models was reduced by about 50% (Fig. 6). The 50% effective concentration (EC_50_) values for BoNT/FA were ~0.07 U/well for the RSC cells and ~0.02 U/well for the human iPSC-derived neurons. In comparison, EC_50_ values for BoNT/A1 are ~0.3 U/well in both cell models, and for BoNT/B1, the values were ~30 and ~15 U/well in RSC cells and hiPSC-derived neurons, respectively (38). These data indicate that BoNT/FA efficiently enters and cleaves VAMP2 in human neurons.

### Pure BoNT/FA is completely neutralized by antibodies raised against BoNT/A1.

Based on the high degree (>90%) of amino acid similarity of BoNT/FA to BoNT/A1 in the receptor binding domain (26), it seemed reasonable to expect that this toxin would be neutralized by antibodies raised against BoNT/A1. However, previous publications on antibody neutralization of BoNT/FA from total culture supernatant of strain IBCA10-7060 or its subculture CDC69016 (therefore also containing BoNT/B2) did not agree whether BoNT/FA could be neutralized by antibodies raised against the seven known BoNT serotypes. An initial study reported no neutralization, although some protection was observed with high antitoxin concentrations (22), while a later study reported complete neutralization (26).

**FIG 7:**
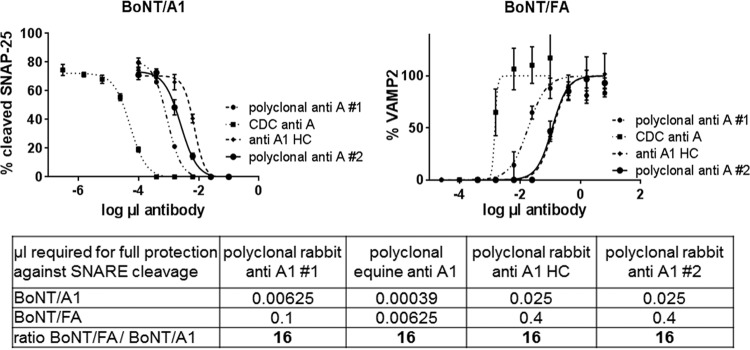
Cell-based analysis of neutralization of BoNT/FA by various polyclonal antibodies raised against BoNT/A1**.** Serial dilutions of antibody and 2.5 pM purified BoNT/FA or BoNT/A1 were preincubated for 1 h prior to exposure to neurons, and hiPSC-derived neurons were exposed to the toxin antibody mixture for 24 h. Cell lysates were analyzed for VAMP2 (relative to syntaxin) or SNAP-25 cleavage by Western blotting. The bands on the Western blot were quantified by densitometry using TotalLab Quant software (Fotodyne), and the percentage of VAMP2 in relation to syntaxin (as the loading control) or the percentage of uncleaved and cleaved SNAP-25 was determined. The graphs were created with Prism6 software using a nonlinear regression analysis and show the average and standard deviation of three replicates. The table under the graphs shows the minimum amount (in microliters) of each antibody required to provide full protection against SNARE cleavage by the two toxins, and the ratio of the antibody required for full protection against BoNT/FA and against BoNT/A1.

The availability of purified BoNT/FA offered quantitative determination of antibody neutralization of this toxin. Two different assays and several different antibodies were employed, a cell-based assay using hiPSC-derived neurons (38), and the mouse neutralization assay. Four different antibodies raised against BoNT/A1 were tested in the cell-based assay. Two antibodies were independently raised against BoNT/A1 toxoid, a third antibody was raised against the HC of BoNT/A1, and the fourth was monovalent antitoxin to BoNT/A1 provided by the Centers for Disease Control and Prevention (CDC) (Atlanta, GA). Western blot analyses showed that hiPSC-derived neurons that had been exposed to mixtures of either 2.5 pM BoNT/FA or 2.5 pM BoNT/A1 and serially diluted antibody were protected by the antibodies against toxin-induced SNARE (soluble *N*-ethylmaleimide-sensitive factor attachment protein receptor) cleavage in a dose-dependent manner (Fig. 7). All antibodies were able to completely neutralize the activity of both toxins, although lower concentrations of antibody were required to neutralize BoNT/A1. The ratio of the antibody concentration required for full protection against BoNT/FA versus BoNT/A1 was about 16 for all antibodies (Fig. 7), indicating that antibodies raised against BoNT/A1 can neutralize BoNT/FA activity but at an efficiency of about 16-fold lowerthan the efficiency for BoNT/A1 in this assay.

For the mouse bioassay, equine polyclonal antibody raised against BoNT/A1 complex was examined. Animals injected with 500 mLD_50_ of BoNT/FA or BoNT/A1 alone all died within 24 h. In a direct comparison, mice injected with 500 mLD_50_ of either BoNT/A1 or BoNT/FA that was preincubated with serial dilutions of anti-A1 antibody had different survival profiles. Mice injected with BoNT/FA toxin survived at antibody dilutions less than 1:200, whereas the mice injected with BoNT/A1 survived at antibody dilutions less than 1:4,000 (Table 2). This indicates that 20-fold-more antibody was required to protect mice against BoNT/FA than for protection against BoNT/A1, which is similar to the results obtained with the cell-based assay.

**TABLE 2:** Mouse neutralization assay using serial dilutions of polyclonal equine anti-A1 antitoxin^a^

Antibody dilution	No. of mice that survived up to 96 h/total no. of mice injected with the following toxin:	
	BoNT/FA	BoNT/A1
100	4/4	
200	4/4	
400	0/4	
800	0/4	
1,000		4/4
1,600	0/4	
2,000		4/4
4,000		4/4
8,000		0/4
16,000		0/4

a The mice were injected with BoNT/FA and BoNT/A1 toxin at 500 mLD_50_ per mouse. Note that all animal deaths occurred within the first 24 h (i.e., no delayed deaths).

Since the LC of BoNT/FA resembles the BoNT/F5 toxin in its amino acid sequence and its catalytic activity, the neutralization by type F antitoxin (raised to BoNT/F1 complex) was assessed as well. Mice injected with a combination of 500 mLD_50_ of BoNT/FA and type F antitoxin in addition to a dilution of type A antitoxin that alone did not protect mice (1:400) had a delay in the onset of symptoms. However, when the type A antitoxin level was further diluted, mice died within 24 h. This indicates that type F antitoxin has some neutralization effect, but it is minimal.

Finally, protection of mice against BoNT/FA by the heptavalent botulinum antitoxin (hBAT) was examined. Animals injected with 500 mLD_50_ of BoNT/FA plus hBAT (0.125 ml per mouse) were 100% protected and exhibited no signs of botulism during the 4-day observation period. This clearly indicates that hBAT is protective against BoNT/FA.

## DISCUSSION

Dual-toxin-producing strains have been isolated from human botulism cases, the environment, and foods (15, 16, 39–41). Recently, a strain producing three BoNTs (A2, F4, and F5) was identified (20, 42); paradoxically, the strain was reported to be strain 84 of the early studies of Giménez and Ciccarelli (43). The significance of production of two or more toxins by *C. botulinum* strains on their virulence as well as evolutionary consequences is currently unclear. Phylogenetic studies have provided strong evidence for horizontal gene transfer of BoNT gene clusters. Apparently certain ecological niches facilitate the horizontal gene transfer of BoNT gene clusters. Dual BoNT toxin formation occurs in group I (proteolytic) but not group II (nonproteolytic) strains of *C. botulinum*. The reason for group I having the propensity to harbor multiple BoNT gene clusters is not clear, although it may be related to this group’s apparent proclivity to mobilize BoNT gene clusters by horizontal gene transfer. It is interesting that the strain analyzed in this study appears to produce both toxins at relatively high levels (see also reference 26), whereas most dual-toxin-producing strains appear to produce significantly more of one toxin activity than the other based primarily on neutralization studies. The regulatory mechanisms resulting in one of the BoNTs being produced in greater quantities than the minor BoNT is an intriguing question but is not understood. The growth temperature of two dual-toxin-producing cultures has been shown to affect the formation of BoNTs F and B (21, 44). Further work is required to understand the genetic and physiological bases for differential BoNT gene expression in dual-toxin producers.

Purification and subsequent characterization of toxins produced by dual-toxin producers are essential to gain further understanding of this occurrence. However, purification is usually difficult in dual-toxin producers, since the BoNTs are similar in structure, molecular weight, and ionic properties. Affinity chromatography using antibodies specific for different serotypes can be used to purify relatively low quantities of BoNTs, and other approaches such as selective binding to receptor proteins or SNARE (soluble *N*-ethylmaleimide-sensitive fusion factor attachment protein receptors) substrates is theoretically feasible, but such approaches would require extensive work. In this study, we used the strategy of genetically inactivating the *bont/*B2 gene in the dual-toxin-producing *C. botulinum* strain CDC69016, resulting in the BoNT/FA toxin being singularly produced in sufficient amounts for isolation of about 1 mg of toxin from 6 liters of culture. This allowed the first reported characterization of this interesting novel BoNT in purified form produced by its native host.

The novel BoNT/FA isolated and characterized here was first described in 2014 as a new serotype, BoNT/H, which is produced by a dual-toxin-producing *C. botulinum* strain isolated from a human infant botulism case (22, 23). The possibility of a new BoNT serotype being produced, which would be resistant to currently available antitoxins and potentially might evade detection methods and diagnostic tests, resulted in considerable concern from a bioterrorism perspective and prompted additional studies of this strain and its toxins. These studies revealed that the toxins produced by this strain were neutralized by heptavalent antitoxin and by antitoxins raised to BoNT/A1 and BoNT/B1, although less efficiently than BoNT/A1 (26). Sequence analysis of the toxin showed that it has a chimeric structure, resembling BoNT/A1 in its receptor binding domain, BoNT/F5 in its catalytic domain (light chain), and BoNT/F1 in its translocation domain (24–26). This F-A composition indicated renaming of this toxin to BoNT/FA, to reflect its chimeric nature, to be consistent with current nomenclature of chimeric toxins for serotypes C and D (27, 28), and to reflect its neutralization by current antitoxins. However, these early studies were undertaken using culture supernatant of the dual-toxin-producing strain, and thus, interference of the second BoNT cannot be excluded even when BoNT/B2 activity is reduced by type B antitoxin. The isolation of pure BoNT/FA toxin described here offered the opportunity to unambiguously characterize this toxin immunologically and biologically.

Interestingly, during the purification procedure of BoNT/FA from strain CDC69016/B2tox^−^, in which the *bont*/B2 gene had been inactivated, only NTNH (nontoxic nonhemagglutinin) remained associated with BoNT/FA after the first ion exchange chromatography, which is utilized to isolate BoNT complexes (Fig. 5A and B). The *bont* genes are arranged in one of two general toxin gene clusters, the *ntnh-ha* cluster or the *ntnh-orfX* cluster (12). In strain IBCA10-7060/CDC69016, the *bont*/B2 gene is arranged in an *ntnh-ha* toxin gene cluster encoding nontoxic nonhemagglutinin and three hemagglutinins (HAs), whereas the BoNT/FA gene is arranged in an *ntnh-orfX* cluster encoding NTNH, P47, and putatively three ORFX proteins (23). It has previously been shown that immunoprecipitation of BoNTs with OrfX gene clusters detected only the full-length NTNH in association with the BoNT but not the OrfX proteins, whereas the BoNTs from HA gene clusters contained the NTNH and HA proteins (45). Thus, the association of BoNT/FA with only NTNH and the elution of the HA proteins in a separate fraction (Fig. 5B) indicates that the BoNT/FA toxin assembles only with the complex proteins from its own toxin gene cluster (*orfX* cluster), even though the complex proteins (NTNH, HAs) of the *bont*/B2 cluster were produced in the absence of BoNT/B2. This was further confirmed by an immunoprecipitation assay using BoNT/A-specific antibodies to capture the BoNT/FA toxin from the CDC69016/B2tox^−^ culture supernatant (Fig. 2B).

Because strain IBCA10-7060/CDC69016 has been described as being most similar to other group I proteolytic *C. botulinum* strains based on genomic analysis (25), and the F and A domains in BoNT/FA are derived from proteolytic strains, where proteolytic activation (“nicking”) of the toxins occurs in culture, it was expected that the BoNTs produced by this strain would be nicked endogenously. However, Western blot analysis of culture supernatant indicated that even after 5 days in culture at 37°C, BoNT/FA and most of BoNT/B2 remained unnicked (Fig. 2A). Analysis of BoNT/FA toxin activity in the CDC69016/B2tox^−^ strain further demonstrated that BoNT/FA was strongly activated by trypsinization, with an ~40-fold increase in toxicity in mice and an at least 20-fold increase in cultured neurons (Fig. 4). These data show that BoNT/FA is not proteolytically activated in its native strain and that toxin activity increases greatly upon proteolytic activation. This highlights the importance of trypsin treatment of culture supernatants when analyzing activity and neutralization of novel BoNTs.

Characterization of the purified BoNT/FA toxin confirmed that this toxin cleaves VAMP2 at the same cleavage site as BoNT/F5 (Fig. 3) (24), which is different from the cleavage site of other BoNT/F subtypes (46). Furthermore, the toxin had an ~5- to 8-fold-lower specific toxicity in mice than BoNT/A1 (Table 1), whereas relative activity in cultured human neurons, based on using equivalent mLD_50_ per well, was about 20-fold greater than the relative activity for BoNT/A1 and almost 1,000-fold greater than that for BoNT/B1 (Fig. 6) (38). The mechanism underlying the higher activity in cultured neurons is currently unknown. Possibilities include species-specific differences in either cell binding/entry or VAMP cleavage, toxin-specific characteristics affecting *in vivo* distribution or elimination, or specific properties of the neuronal cultures used in this project. Further research exploring this unique characteristic of BoNT/FA will be required to determine any potential effects on the activity of this toxin in humans.

Finally, neutralization studies in mice and cultured human neurons both indicated that purified BoNT/FA activity was completely neutralized by various type A antitoxins as well as by multivalent antitoxin raised against the seven BoNT serotypes. Neutralization for all of the antibodies tested was about 16- to 20-fold less effective than for BoNT/A1 (Fig. 7). Variable antitoxin neutralization efficiency of subtypes within a BoNT serotype has previously been observed (47, 48) and is not surprising, considering that the antitoxins are raised against one subtype of each serotype. Neutralization by various antitoxins against BoNT/A1 presented here support classification of this toxin as a chimeric BoNT/FA toxin rather than a new serotype. It will be interesting to evaluate neutralization by antibodies raised against purified BoNT/F5, although this domain comprises the light chain which is generally less antigenic than the heavy chain. Regardless of the name of this toxin, our data demonstrate that this toxin can be neutralized by currently available countermeasures. In addition, this toxin has some unique characteristics, and future studies exploring these in more detail will be of considerable interest in helping to increase our understanding of the expanding repertoire of botulinum neurotoxins.

## MATERIALS AND METHODS

### Biosafety and biosecurity.

All laboratories and personnel are registered with the Federal Select Agent Program for research involving botulinum neurotoxins (BoNTs) and BoNT-producing strains of clostridia. The research program, procedures, documentation, security, and facilities are closely monitored by the University of Wisconsin−Madison Biosecurity Task Force, the University of Wisconsin−Madison Office of Biological Safety, the University of Wisconsin Select Agent Program, and the Centers for Disease Control and Prevention (CDC) and the Animal and Plant Health Inspection Service (APHIS) as part of the University of Wisconsin−Madison and CDC Select Agent Program. All personnel have undergone suitability assessments and completed rigorous and continuing biosafety training, including biosafety level 3 (BSL3) or BSL2 and select agent practices, before participating in laboratory studies involving BoNTs and neurotoxigenic *C. botulinum*. All animal experiments have been approved by the University of Wisconsin or CDC IACUC.

### Antibodies.

Anti-BoNT/A1 and anti-BoNT/B1 antibodies were prepared as previously described (49). Anti-BoNT/FA antibodies were generated as follows. BoNT/FA toxoid was prepared by dialyzing purified BoNT/FA against 0.05 M sodium phosphate buffer (pH 7.5) containing 0.15 M NaCl and 0.5% formaldehyde for 14 days at room temperature. The toxoid was then dialyzed against phosphate-buffered saline (PBS) for 48 h to remove formaldehyde. Lack of toxicity in the BoNT/FA toxoid preparation was confirmed by injection of 1 µg of toxoid/mouse into two mice. A female New Zealand White rabbit was injected both subcutaneously and intramuscularly with a total of 250 µg of toxoid mixed with an equal volume of aluminum hydroxide adjuvant followed by a boost with 80 µg toxoid in adjuvant on day 30 and two boosts with ~100 µg toxoid at ~30-day intervals without adjuvant. Serum obtained from a central ear artery bleed of the immunized rabbit was fractionated by protein A chromatography to obtain polyclonal anti-BoNT/FA IgG.

### Bacterial strains and growth conditions.

*C. botulinum* strain CDC69016 (26) (originally named strain IBCA10-7060) (22, 23) was obtained from the Centers for Disease Control and Prevention (Atlanta, Ga). This strain was originally isolated from an infant botulism case by the California Department of Public Health and designated IBCA10-7060 (22). It was transferred to the CDC, where it was subcultured and stored under the name CDC69016 (26). The draft genome sequence of this strain under the name CFSAN024410 is available in GenBank accession no. JSCF00000000.1 (25). *C. botulinum* cultures were grown at 37°C in TYG medium (3% Bacto tryptone, 2% yeast extract, 0.1% sodium thioglycolate [pH 7.3]) for isolation of mutant clones, in TPGY medium (5% Trypticase peptone, 0.5% Bacto peptone, 0.4% glucose, 2% yeast extract, 0.1% l-cysteine [pH 7.4]) for strain characterization and maintenance, or in BoNT/A1 toxin production medium (TPM) (2% casein hydrolysate [NZ Case TT] 1% yeast extract, 0.5% glucose [pH 7.2]) for production and characterization of the BoNT. NZ Case TT was from Kerry Bio-Science (Beloit, WI); all other bacterial medium components and chemicals were purchased from Becton Dickinson Microbiology Systems (Sparks, MD) and Sigma-Aldrich (St. Louis, MO). Cultures were kept in TPGY medium with 20% glycerol for long-term storage at −80°C. *Escherichia coli* strain DH10B (Life Technologies, Carlsbad, CA) was used for the cloning and maintenance of the ClosTron retargeting construct. *E. coli* strain CA434 served as a donor for conjugal transfer of the retargeting construct from *E. coli* into *C. botulinum*.

Antibiotics were used at the following concentrations in *C. botulinum* cultures: cycloserine at 250 µg/ml, thiamphenicol at 15 µg/ml, and erythromycin at 30 µg/ml. In *E. coli* cultures, ampicillin at 100 µg/ml and chloramphenicol at 25 µg/ml in agar plates and 12.5 µg/ml in broth were used. Clostridial cultures were handled under anaerobic conditions inside an anaerobic chamber (model 1025; Forma Anaerobic System, Marietta, OH), with an initial gas mixture comprised of 80% N_2_, 10% CO_2_, and 10% H_2_. The chamber’s vacuum pump was equipped with an exhaust filter (Balston model CV-0118-30; Parker Hannifin Corp., Haverhill, MA) to prevent release of clostridial spores into the laboratory. Glass culture tubes for the growth of clostridia were flushed with nitrogen and sealed with butyl rubber septum stoppers (Bellco Glass, Vineland, NJ) prior to sterilization. Resazurin (2 µg/ml) was added to TYG agar as an indicator of anaerobic conditions, and plates were prereduced by overnight incubation in the anaerobic chamber prior to use.

### Construction of *bont*/B2 insertional mutant strain.

The botulinum neurotoxin B2 gene (NZ45_18945; GenBank accession number JSCF01000105) in strain CDC69016 was inactivated with the ClosTron mutagenesis system by insertion of a mobile group II intron between nucleotides 381 and 382 on the sense strand using plasmid pMTL007C-E2::Cbo:bontbvB-381s as previously described (33). *C. botulinum* clones harboring the ClosTron plasmid were selected for on media containing both cycloserine and thiamphenicol. Individual thiamphenicol-resistant colonies were restreaked on media containing cycloserine and erythromycin (Erm) for isolation of intron integrants. Next, erythromycin-resistant clones were screened for sensitivity to thiamphenicol to confirm loss of the intron vector. One Erm^r^ Th^s^ clone was selected and designated CDC69016/B2tox^−^. The clone underwent four rounds of colony purification on plates containing erythromycin to ensure purity. Last, a culture was heated for 10 min at 80°C and plated on media with and without Erm to ensure absence of growth, indicating that no spores of either the wild-type strain or mutant were present.

### Analysis of the BoNT/B2tox^−^ strain by PCR, sequencing, and Southern hybridization analysis.

Genomic DNA was isolated from the wild-type *C. botulinum* strain CDC69016 and the CDC69016/B2tox^−^ mutant using the ChargeSwitch genomic DNA (gDNA) Mini Bacteria kit (Life Technologies, Carlsbad, CA) following the manufacturer’s instructions. Specific primers B268F (F stands for forward) and B506R (R stands for reverse) (see Table S1 in the supplemental material) were used to amplify a region of the *bont*/B2 gene flanking the ClosTron insertion site. PCR primers were purchased from Integrated DNA Technologies, Inc. (Coralville, IA). PCR amplifications were performed using Phusion High-Fidelity PCR master mix with H F buffer (New England Biolabs Inc., Ipswich, MA), 0.5 µM primers and ~250 ng of genomic DNA in a GeneAmp PCR system 9700 (Applied Biosystems, Foster City, CA). PCR fragments were treated with ExoSAP (Affymetrix, Santa Clara, CA) to remove PCR primers, and sequencing reactions were performed using the BigDye Terminator v1.1 Cycle Sequencing kit (Applied Biosystems, Foster City, CA), and the *bont*/B2-specific PCR primers (Table S1). The sequencing reactions were analyzed at the University of Wisconsin Biotechnology Center, and the nucleotide sequences were analyzed using MacVector software (MacVector, Inc., Cary, NC).

10.1128/mSphere.00100-15.1 Table S1  Oligonucleotide primers used in this study Download Table S1, DOCX file, 0.1 MB. Copyright © 2016 Pellett et al. 2016 Pellett et al. This content is distributed under the terms of the Creative Commons Attribution 4.0 International license.

To confirm that only a single intron insertion had occurred in the mutant clone, 5 µg of the genomic DNA from the wild-type strain CDC69016 and the CDC69016/B2tox^−^ mutant strain were digested with restriction endonuclease HindIII and analyzed by standard Southern hybridization (50) using 2 × 10^6^ cpm/ml ^32^P-labeled Erm gene probe. The 737-bp DNA fragment containing the coding region of the *ermBP* was prepared by PCR amplification using primers ErmF and ErmR (see Table S1 in the supplemental material) and plasmid pJIR1457 (GenBank accession number U90555) as a template. After hybridization and washing, the membrane was exposed to a Classic Blue autoradiography film BX (Molecular Technologies, St. Louis, MO) with a BioMax intensifying screen (Eastman Kodak, Rochester, NY) for 6 to 24 h at −80°C.

### Mass spectrometry of toxin and its activity.

One nanogram of BoNT/FA was added to peptide substrate LSELDDRADALQAGASQFESSAAKLKRKYWWKNLK (SubB) in the presence of reaction buffer as described previously (24). Another aliquot of 1 ng of BoNT/FA was added to another peptide substrate TSNRRLQQTQAQVDEVVDIMRVNVDKVLERDQKLSELDDRADAL (SubF) also in the presence of reaction buffer. Following a 4-h incubation at 37°C, a 2-µl aliquot of each reaction supernatant was mixed with 18 µl of matrix solution, and a 0.5-µl aliquot of this mixture was pipetted onto one spot of a MALDI plate as described previously (24). Mass spectra of each spot were obtained by scanning from 900 to 5500 *m/z* in MS-positive ion reflector mode on an Applied Biosystems 5800 proteomics analyzer (Framingham, MA). The instrument uses an Nd-YAG laser at 355 nm, and each spectrum is an average of 2,400 laser shots.

To positively identify BoNT/FA via its amino acid sequence, 1 µg of the toxin was added to 13 µl of 100 mM ammonium bicarbonate at pH 7.5 and 2 µl of trypsin at 0.5 mg/ml in water. Following mixing, the mixture was heated at 52°C for 10 min, followed by the addition of 1 µl of 10% of trifluoroacetic acid. Peptides from the toxin were identified by LC-MS/MS with database searching as described previously (20).

### Analysis of protein production by the ClosTron clones by Western blotting, immunoprecipitation, and cell-based neutralization analyses.

The CDC69016/B2tox^−^ mutant was screened for its ability to produce BoNTs B2 and FA. CDC69016/B2tox^−^ was cultured in TPGY medium at 37°C for 72 h. Total culture (including cells and supernatant) was denatured by the addition of NuPAGE SDS sample buffer (Life Technologies, Carlsbad, CA) and β-mercaptoethanol (Bio-Rad, Hercules, CA) to 1×, and the samples were heated for 5 min at 95°C. Cell lysates were analyzed by Western blotting with polyclonal affinity-purified rabbit IgG specific to BoNT/B1 or BoNT/A1 prepared in our laboratory (49) and a bovine anti-rabbit secondary antibody (Santa Cruz Biotechnology, Dallas, TX). Images were obtained using a chemiluminescent PhosphaGLO alkaline phosphatase (AP) substrate (KPL, Gaithersburg, MD) and Fotodyne, Inc. FOTO/Analyst/FX imaging system (Hartland, WI).

For immunoprecipitation analysis, culture supernatants of strain CDC69016/B2tox^−^ were analyzed as described previously (45). Briefly**,** polyclonal antibodies raised against BoNT/A1 or BoNT/B1 (49) were used for immunoprecipitation of the respective toxins from 1 ml of culture supernatants. The recovered proteins were analyzed by SDS-PAGE using 4 to 12% Bis-Tris NuPAGE Novex gels in morpholineethanesulfonic acid (MES) running buffer (Life Technologies, Carlsbad, CA). Each sample was analyzed under nonreduced (no dithiothreitol [DTT] added) and reduced (100 mM DTT) conditions. The gels were stained using Coomassie blue, and SeeBlue Plus2 prestained protein standards (Life Technologies, Carlsbad, CA) were used to estimate the molecular mass of detected proteins.

In order to confirm the absence of BoNT/B production in the CDC69016/B2tox^−^ strain, a cell-based neutralization assay was performed. For this, 5 µl of CDC69016/B2tox^−^ trypsinized culture supernatant was incubated with 2 µl of rabbit polyclonal anti-BoNT/A1 or anti-BoNT/B1 antibodies (each estimated to contain approximately 100 IU/ml of activity by mouse protection assay and cell-based assays) (38, 51, 52), a combination of both antibodies, or no antibodies in 100 µl of culture media for 1 h. The toxin/antibody mixtures were then added to the iCell neurons for 24 h (for details on cell culture, see section on cell-based assays below). Cells were harvested by lysis in lithium dodecyl sulfate (LDS) sample buffer (Life Technologies), and cell lysates were analyzed by Western blotting as described below. Graphs were prepared using Microsoft Excel.

### Purification of BoNT/FA.

Freshly prepared 11.5 liters of TPM (~1.65 liters in seven 2-liter bottles) were inoculated 1:1,000 with an actively growing culture of *C. botulinum* strain CDC69016/B2tox^−^. Culture bottles were incubated statically for 120 h at 37°C inside the anaerobic chamber. The cultures were chilled for 1 h in an ice-water bath, followed by lowering the pH of the cultures to 3.5 by the slow addition of 3 N H_2_SO_4_. The precipitate formed was collected by centrifugation at 15,000 × *g* and 4°C for 10 min. The pellet was washed once with distilled water, and the precipitate was collected by centrifugation as described above. The toxin was extracted from the pellet two times by suspension in 800 ml (400 ml for the second extraction) of 0.1 M sodium citrate buffer (pH 5.5) with gentle stirring for 2 h at ambient temperature followed by centrifugation. The proteins from both extraction steps were precipitated by gradually adding solid ammonium sulfate to 60% saturation (39 g/100 ml), and the suspension was stored at 4°C.

The ammonium sulfate-precipitated material from both extractions was combined and collected by centrifugation at 15,000 × *g* and 4°C for 20 min. The pellet was resuspended in 100 ml of 50 mM sodium phosphate buffer (pH 6.0), RNase A was added to a final concentration of 100 µg/ml, and the suspension was incubated for 3 h at 37°C. The extract or RNase A digestion product was centrifuged at 15,000 × *g* for 20 min at 20°C to remove insoluble material, and solid ammonium sulfate was added gradually to the supernatant until 60% saturated (39 g/100 ml) and held at 4°C for further processing. The precipitated material was collected by centrifugation at 15,000 × *g* and 4°C for 20 min, and the pellet was dissolved in 0.1 M sodium phosphate buffer (pH 6.0). Trypsin [l-(tosylamido-2-phenyl)ethyl chloromethyl ketone (TPCK)-treated trypsin; Worthington Biochemical Corp., Lakewood, NJ] was added to a final concentration of 20 µg/ml, and the solution was incubated for 60 min at 37°C. Soybean trypsin inhibitor (Sigma type II-S [soybean]; Sigma-Aldrich, St. Louis, MO) was added to the solution to a final concentration of 80 µg/ml and incubated for 10 min at ambient temperature to inactivate trypsin. The trypsin-treated solution was precipitated by the addition of solid ammonium sulfate at 39 g/100 ml and stored at 4°C.

For isolation of the 150-kDa BoNT/FA, half of the RNase A- and trypsin-treated ammonium sulfate precipitate was collected by centrifugation at 15,000 × *g* and 4°C for 20 min, resuspended in 10 ml of 0.05 M sodium citrate buffer (pH 5.5), and dialyzed for 4 h at room temperature with three dialysis changes at 1-h intervals. The dialyzed solution was centrifuged at 15,000 × *g* and 20°C for 20 min to remove insoluble material, and the supernatant was loaded onto a 260-ml DEAE-Sephadex A-50 column (2.5 cm by 53 cm) (Sigma-Aldrich) equilibrated with 50 mM sodium citrate buffer (pH 5.5) at room temperature. About 1.5 column volumes of 50 mM sodium citrate buffer (pH 5.5) were added, and eluted fractions were monitored at an optical density at 278 nm (OD_278_) and analyzed by Coomassie blue-stained SDS-PAGE. The unbound fractions from the ascending portion of the second peak containing the crude toxin complex were pooled and precipitated by the addition of solid ammonium sulfate to 39 g/100 ml and stored at 4°C. The precipitated BoNT/FA complex from the DEAE chromatography was collected by centrifugation at 15,000 × *g* and 4°C for 20 min, and the pellet was dissolved in 0.1 M sodium phosphate buffer (pH 6.0). The concentration of the BoNT/FA complex in the solution was estimated by measuring *A*_278_ and using an extinction coefficient of 1.63.

To ensure complete proteolytic processing of the BoNT/FA, a second round of trypsin treatment was performed. Trypsin was added at a 30:1 ratio (FA complex to trypsin) and incubated at 37°C for 30 min. Soybean trypsin inhibitor was added at a ratio of 4:1 (trypsin inhibitor to trypsin), and the solution was then precipitated with ammonium sulfate (0.39 g/ml). The ammonium sulfate-precipitated trypsinized toxin complex was collected by centrifugation at 15,000 × *g* and 4°C for 20 min and resuspended in 8 ml of 0.01 M sodium phosphate buffer (pH 7.6). The solution was dialyzed at room temperature with three dialysis changes at 1-h intervals and loaded onto a DEAE-Sephadex column (0.9 cm by 10 cm) (Sigma-Aldrich) equilibrated with 0.01 M sodium phosphate buffer (pH 7.6). The column was washed with the same buffer until the *A*_278_ reached 0, and a 100-ml linear gradient from 0 mM to 350 mM NaCl in 0.01 M sodium phosphate buffer (pH 7.6) was then applied. Fractions were monitored at *A*_278_ and analyzed by SDS-PAGE. The fractions containing ~95% pure toxin (BoNT/FA) were pooled and precipitated by the addition of ammonium sulfate to 39 g/100 ml. The suspension containing the precipitated toxin was centrifuged as described above at 4°C, and the pellet was dissolved in 0.02 M sodium phosphate buffer (pH 7.5) containing 0.1 M NaCl. The toxin solution was filtered through a 0.22-µm syringe filter (Millex-GV; Millipore), and the OD_278_ was measured. The toxin concentration was estimated using an extinction coefficient of 1.63 at 278 nm. Glycerol was added to a final concentration of 40%, and the toxin solution was stored at −20°C.

### Mouse bioassay.

Toxicity determinations of culture supernatants and purified toxin were conducted by standard mouse bioassay as previously described (53, 54). For toxicity determinations of culture supernatants, the supernatants from two separate batches of the CDC69016/B2tox^−^ strain grown for 6 days in TPM were passed through a 0.2-μm Nalgene polyethersulfone (PES) filter, and a portion of the filtered culture supernatant was treated with 20 µg/ml trypsin (TPCK-treated trypsin; Worthington Biochemical Corp., Lakewood, NJ) for 60 min at 37°C. Trypsin inhibitor (Sigma type II-S [soybean]; Sigma-Aldrich, St. Louis, MO) was added at a 4:1 ratio (trypsin inhibitor to trypsin). The trypsinized or untrypsinized culture supernatants were diluted as indicated in the Results section and figures in 0.03 M sodium phosphate buffer (pH 6.3) containing 0.2% gelatin (GelPhos buffer). For purified BoNT, serial dilutions were prepared in GelPhos buffer. Six groups of 4 mice were injected intraperitoneally with 0.5 ml per mouse with the toxin dilutions, respectively, and mice were observed for 6 days. The mouse LD_50_/ml (mLD_50_/ml) of the culture supernatants were determined based on the method of Reed and Muench (55). One mouse LD_50_ (mLD_50_) is the quantity of toxin needed to achieve 50% deaths of injected mice in this assay.

For the neutralization assay, BoNT/FA or BoNT/A1 was diluted to 1,000 mLD_50_/ml in GelPhos buffer (56), and neutralization by an equine monovalent research antitoxin (Auburn University, Auburn, AL; raised using Metabiologics [Madison, WI] complex toxins A 2,623 IU/ml) was determined. The antitoxin was diluted in GelPhos buffer, and 0.5 ml of each dilution was mixed with 2 ml of diluted toxin. The toxin-antitoxin mixtures were incubated at ambient temperature for 30 min, and then groups of four mice were exposed by intraperitoneal (i.p.) injection (0.5 ml/mouse). The effect of hBAT was similarly tested by adding 0.25 ml of hBAT to 1,000 mLD_50_ per ml BoNT/FA. The effect of serotype F antitoxin (Auburn University, Auburn, AL; raised using Metabiologics [Madison, WI] complex toxins F1, 996 IU/ml) was assessed by combining 124 IUs of serotype F antitoxin with a dilution of serotype A antitoxin beyond 100% protection (1:400 or 1:800, as indicated in Results) and 1,000 mLD_50_ per ml BoNT/FA. The toxin-antitoxin mixtures were incubated and injected into mice as described above. Mice were observed for signs of botulism for 4 days (56). The mouse neutralization studies were conducted according to protocols approved by the CDC Institutional Animal Care and Use Committee.

### Cell-based toxicity and neutralization assay.

Cell-based assays were performed essentially as previously described (38). Human induced pluripotent stem cell (hiPSC)-derived neurons (iCell neurons) were purchased from Cellular Dynamics International (CDI) (Madison, WI) and were seeded into 96-well TPP plates (Techno Plastic Products, Midwest Scientific, Valley Park, MO) that had been coated with 0.01% poly-l-ornithine and 8.3 µg/cm^2^ Matrigel (BD Biosciences, East Rutherford, NJ) at a density of about 35,000 to 40,000 cells per well. The cells were maintained in the provided culture medium per the company instructions. Five to 7 days after seeding, the cells were used for the toxin activity and neutralization assays. The primary rat spinal cord (RSC) cells were prepared from embryonic day 15 (E15) Sprague Dawley rat pups and seeded into 96-well TPP plates that had been coated with 0.01% poly-l-ornithine and 8.3 µg/cm^2^ Matrigel at a density of 50,000 cells/well. RSC cells were maintained in Neurobasal medium supplemented with B27, GlutaMAX, and penicillin-streptomycin (all from Life Technologies) as previously described (52, 57) and used for the toxin assay after 19 days in culture.

For the toxin activity assay, serial dilutions of purified BoNT/FA were prepared in the respective cell culture medium. The cells were exposed to the serial toxin dilutions in 50 µl of culture medium for 48 h at 37°C and 5% CO_2_. The toxin was aspirated from the cells, and cell lysates were prepared in 50 µl of lithium dodecyl sulfate sample buffer (Life Technologies). For the cell entry assay, iCell neurons were exposed to 2 U of BoNT/FA in 50 µl of culture medium (6.7 pM), and cells were harvested by lysis in 50 µl of LDS sample buffer as indicated. The cell lysates were analyzed by Western blotting for VAMP2 cleavage as previously described (52, 57). Images were obtained using PhosphaGlo reagent (KPL, Gaithersburg, MD) and a Fotodyne/FOTO/Analyst FX imaging system (Harland, WI), and the VAMP2 signal was analyzed in relation to the syntaxin signal (loading control) by densitometry using TotalLab Quant software (Fotodyne, Harland, WI). EC_50_ values were estimated using GraphPad Prism 6 software and a nonlinear regression analysis, variable slope, and four parameters.

In order to determine the effect of trypsin on the BoNT/FA activity in neuronal cells, the trypsinized and untrypsinized CDC69016/B2tox^−^ culture supernatants (prepared as for the mouse bioassay described above) were filtered through a 0.2-μm Acrodisc. Serial dilutions of each culture supernatant were prepared in culture media, and iCell neurons were exposed to these serial dilutions for 48 h. Cells were harvested by lysis in LDS sample buffer (Life Technologies) and analyzed as described above.

For the antibody neutralization assay, four different antibodies raised against BoNT/A1 were analyzed. Three of these antibodies were prepared in our laboratories in rabbits immunized with BoNT/A1 toxoid (anti-A1 antibodies 1 and 2), or with BoNT/A1 heavy chain (anti-A1 HC), and the fourth antibody was derived from a horse immunized with BoNT/A1 (CDC, Atlanta, GA). BoNT/A1 and BoNT/FA at 2.5 pM were combined with the indicated amounts of polyclonal rabbit anti-A1 antibody 1, polyclonal rabbit anti-A1 antibody 2, polyclonal horse anti-A1 antibody, and polyclonal rabbit anti-A1 HC in culture media, and incubated for 1 h at 37°C. The toxin/antibody mixtures were then added to iCell neurons in triplicate (50 µl/well), and incubated for 24 h at 37°C and 5% CO_2_. Cell lysates were prepared in 50 µl of LDS sample buffer (Life Technologies) and analyzed as described above. To compare neutralization of BoNT/A1 and BoNT/FA, the ratio of the 50% inhibitory concentration (IC_50_) values for BoNT/A1 and BoNT/FA was determined for each antibody.

## References

[1] Rossetto O, Pirazzini M, Montecucco C 2014 Botulinum neurotoxins: genetic, structural and mechanistic insights. Nat Rev Microbiol 12:535–549. doi:10.1038/nrmicro3295. 24975322

[2] Johnson EA, Montecucco C 2008 Chapter 11. Botulism, p 333–368. In Engel AWG (ed), Handbook of clinical neurology, vol 91 Neuromuscular junction disorders. Elsevier, Amsterdam, The Netherlands.

[3] Centers for Disease Control and Prevention. 2010 Investigational heptavalent botulinum antitoxin (HBAT) to replace licensed botulinum antitoxin AB and investigational botulinum antitoxin E. MMWR Morb Mortal Wkly Rep 59:299. 20300057

[4] Arnon SS 1980 Infant botulism. Annu Rev Med 31:541–560. doi:10.1146/annurev.me.31.020180.002545. 6772092

[5] Arnon SS, Schechter R, Maslanka SE, Jewell NP, Hatheway CL 2006 Human botulism immune globulin for the treatment of infant botulism. N Engl J Med 354:462–471. doi:10.1056/NEJMoa051926. 16452558

[6] Arnon SS, Schechter R, Inglesby TV, Henderson DA, Bartlett JG, Ascher MS, Eitzen E, Fine AD, Hauer J, Layton M, Lillibridge S, Osterholm MT, O’Toole T, Parker G, Perl TM, Russell PK, Swerdlow DL, Tonat K, Working Group on Civilian Biodefense 2001 Botulinum toxin as a biological weapon: medical and public health management. JAMA 285:1059–1070. 11209178 10.1001/jama.285.8.1059

[7] Balali-Mood M, Moshiri M, Etemad L 2013 Medical aspects of bio-terrorism. Toxicon 69:131–142. doi:10.1016/j.toxicon.2013.01.005. 23339855

[8] Peck MW, Stringer SC, Carter AT 2011 *Clostridium botulinum* in the post-genomic era. Food Microbiol 28:183–191. doi:10.1016/j.fm.2010.03.005. 21315972

[9] Hill KK, Smith TJ, Helma CH, Ticknor LO, Foley BT, Svensson RT, Brown JL, Johnson EA, Smith LA, Okinaka RT, Jackson PJ, Marks JD 2007 Genetic diversity among botulinum neurotoxin-producing clostridial strains. J Bacteriol 189:818–832. doi:10.1128/JB.01180-06. 17114256 PMC1797315

[10] Gimenez DF, Gimenez JA 1992 Serological subtypes of botulinum neurotoxins, p 421–431. In DasGupta BR (ed), Botulinum and tetanus neurotoxins: neurotransmission and biomedical aspects. Plenum Press, New York, NY.

[11] Hatheway CL, Johnson EA 1998 *Clostridium*: the spore bearing anaerobes, p 731–782. In Collier L, Ballows A, Sussman M (ed), Topley and Wilson’s microbiology and microbial infections, vol 2, 9th ed. Arnold, London, United Kingdom.

[12] Hill KK, Smith TJ 2013 Genetic diversity within *Clostridium botulinum* serotypes, botulinum neurotoxin gene clusters and toxin subtypes. Curr Top Microbiol Immunol 364:1–20. doi:10.1007/978-3-642-33570-9_1. 23239346

[13] Montecucco C, Rasotto MB 2015 On botulinum neurotoxin variability. mBio 6:e02131-14. doi:10.1128/mBio.02131-14. 25564463 PMC4313909

[14] Hatheway CL, McCroskey LM, Lombard GL, Dowell VR Jr. 1981 Atypical toxin variant of *Clostridium botulinum* type B associated with infant botulism. J Clin Microbiol 14:607–611. 7037830 10.1128/jcm.14.6.607-611.1981 PMC274006

[15] Giménez DF 1984 *Clostridium botulinum* subtype Ba. Zentralbl Bakteriol Mikrobiol Hyg A 257:68–72. 6380157

[16] Giménez DF, Giménez JA 1983 Identification of strain B 657 of *Clostridium botulinum*. Rev Argent Microbiol 15:51–55. 6400761

[17] Ciccarelli AS, Gimenez DF 1971 Serological study of *Clostridium botulinum* type A and strain 84 toxins. Rev Latinoam Microbiol 13:67–74. 4941891

[18] Franciosa G, Ferreira JL, Hatheway CL 1994 Detection of type A, B, and E botulism neurotoxin genes in *Clostridium botulinum* and other *Clostridium* species by PCR: evidence of unexpressed type B toxin genes in type A toxigenic organisms. J Clin Microbiol 32:1911–1917. 7989542 10.1128/jcm.32.8.1911-1917.1994 PMC263902

[19] Hutson RA, Zhou Y, Collins MD, Johnson EA, Hatheway CL, Sugiyama H 1996 Genetic characterization of *Clostridium botulinum* type A containing silent type B neurotoxin gene sequences. J Biol Chem 271:10786–10792. doi:10.1074/jbc.271.18.10786. 8631890

[20] Kalb SR, Baudys J, Smith TJ, Smith LA, Barr JR 2014 Three enzymatically active neurotoxins of *Clostridium botulinum* strain Af84: BoNT/A2, /F4, and /F5. Anal Chem 86:3254–3262. doi:10.1021/ac5001509. 24605815 PMC4522913

[21] Barash JR, Arnon SS 2004 Dual toxin-producing strain of *Clostridium botulinum* type Bf isolated from a California patient with infant botulism. J Clin Microbiol 42:1713–1715. doi:10.1128/JCM.42.4.1713-1715.2004. 15071029 PMC387584

[22] Barash JR, Arnon SS 2014 A novel strain of *Clostridium botulinum* that produces type B and type H botulinum toxins. J Infect Dis 209:183–191. doi:10.1093/infdis/jit449. 24106296

[23] Dover N, Barash JR, Hill KK, Xie G, Arnon SS 2014 Molecular characterization of a novel botulinum neurotoxin type H gene. J Infect Dis 209:192–202. doi:10.1093/infdis/jit450. 24106295

[24] Kalb SR, Baudys J, Raphael BH, Dykes JK, Lúquez C, Maslanka SE, Barr JR 2015 Functional characterization of botulinum neurotoxin serotype H as a hybrid of known serotypes F and A (BoNT F/A). Anal Chem 87:3911–3917. doi:10.1021/ac504716v. 25731972 PMC4522910

[25] Gonzalez-Escalona N, Thirunavukkarasu N, Singh A, Toro M, Brown EW, Zink D, Rummel A, Sharma SK 2014 Draft genome sequence of bivalent *Clostridium botulinum* strain IBCA10-7060, encoding botulinum neurotoxin B and a new FA mosaic type. Genome Announc 2(6):e01275-14. doi:10.1128/genomeA.01275-14. PMC4263833 25502671

[26] Maslanka SE, Lúquez C, Dykes JK, Tepp WH, Pier CL, Pellett S, Raphael BH, Kalb SR, Barr JR, Rao A, Johnson EA 2016 A novel botulinum neurotoxin, previously reported as serotype H, has a hybrid-like structure with regions of similarity to the structures of serotypes A and F and is neutralized with serotype A antitoxin. J Infect Dis 213:379–385. doi:10.1093/infdis/jiv327. 26068781 PMC4704661

[27] Nakamura K, Kohda T, Seto Y, Mukamoto M, Kozaki S 2013 Improved detection methods by genetic and immunological techniques for botulinum C/D and D/C mosaic neurotoxins. Vet Microbiol 162:881–890. doi:10.1016/j.vetmic.2012.11.009. 23206412

[28] Moriishi K, Koura M, Abe N, Fujii N, Fujinaga Y, Inoue K, Ogumad K 1996 Mosaic structures of neurotoxins produced from *Clostridium botulinum* types C and D organisms. Biochim Biophys Acta 1307:123–126. doi:10.1016/0167-4781(96)00006-1. 8679691

[29] Pier CL, Tepp WH, Bradshaw M, Johnson EA, Barbieri JT, Baldwin MR 2008 Recombinant holotoxoid vaccine against botulism. Infect Immun 76:437–442. doi:10.1128/IAI.00843-07. 17967862 PMC2223665

[30] Bradshaw M, Tepp WH, Whitemarsh RC, Pellett S, Johnson EA 2014 Holotoxin activity of botulinum neurotoxin subtype A4 originating from a nontoxigenic *Clostridium botulinum* expression system. Appl Environ Microbiol 80:7415–7422. doi:10.1128/AEM.01795-14. 25239905 PMC4249187

[31] Moreira GM, Cunha CE, Salvarani FM, Gonçalves LA, Pires PS, Conceição FR, Lobato FC 2014 Production of recombinant botulism antigens: a review of expression systems. Anaerobe 28:130–136. doi:10.1016/j.anaerobe.2014.06.003. 24930432

[32] Bradshaw M, Marshall KM, Heap JT, Tepp WH, Minton NP, Johnson EA 2010 Construction of a nontoxigenic *Clostridium botulinum* strain for food challenge studies. Appl Environ Microbiol 76:387–393. doi:10.1128/AEM.02005-09. 19933346 PMC2805223

[33] Marshall KM, Bradshaw M, Johnson EA 2010 Conjugative botulinum neurotoxin-encoding plasmids in *Clostridium botulinum*. PLoS One 5:e11087. doi:10.1371/journal.pone.0011087. 20552020 PMC2884020

[34] Heap JT, Kuehne SA, Ehsaan M, Cartman ST, Cooksley CM, Scott JC, Minton NP 2010 The ClosTron: mutagenesis in *Clostridium* refined and streamlined. J Microbiol Methods 80:49–55. doi:10.1016/j.mimet.2009.10.018. 19891996

[35] Heap JT, Pennington OJ, Cartman ST, Carter GP, Minton NP 2007 The ClosTron: a universal gene knockout system for the genus *Clostridium*. J Microbiol Methods 70:452–464. doi:10.1016/j.mimet.2007.05.021. 17658189

[36] Knox JN, Brown WP, Spero L 1970 The role of sulfhydryl groups in the activity of type A botulinum toxin. Biochim Biophys Acta 214:350–354. doi:10.1016/0005-2795(70)90012-7. 4925645

[37] Yamasaki S, Baumeister A, Binz T, Blasi J, Link E, Cornille F, Roques B, Fykse EM, Südhof TC, Jahn R 1994 Cleavage of members of the synaptobrevin/VAMP family by types D and F botulinal neurotoxins and tetanus toxin. J Biol Chem 269:12764–12772. 8175689

[38] Whitemarsh RC, Strathman MJ, Chase LG, Stankewicz C, Tepp WH, Johnson EA, Pellett S 2012 Novel application of human neurons derived from induced pluripotent stem cells for highly sensitive botulinum neurotoxin detection. Toxicol Sci 126:426–435. doi:10.1093/toxsci/kfr354. 22223483 PMC3307606

[39] Giménez DF, Ciccarelli AS 1978 New strains of *Clostridium botulinum* subtype Af. Zentralbl Bakteriol Orig A 240:215–220. 77604

[40] Franciosa G, Hatheway CL, Aureli P 1998 The detection of a deletion in the type B neurotoxin gene of *Clostridium botulinum* A(B) strains by a two-step PCR. Lett Appl Microbiol 26:442–446. doi:10.1046/j.1472-765X.1998.00367.x. 9717316

[41] Kirma N, Ferreira JL, Baumstark BR 2004 Characterization of six type A strains of *Clostridium botulinum* that contain type B toxin gene sequences. FEMS Microbiol Lett 231:159–164. doi:10.1016/S0378-1097(03)00911-X. 14987759

[42] Dover N, Barash JR, Hill KK, Davenport KW, Teshima H, Xie G, Arnon SS 2013 *Clostridium botulinum* strain Af84 contains three neurotoxin gene clusters: bont/A2, bont/F4 and bont/F5. PLoS One 8:e61205. doi:10.1371/journal.pone.0061205. 23637798 PMC3625220

[43] Giménez DF, Ciccarelli AS 1970 Studies on strain 84 of *Clostridium botulinum*. Zentralbl Bakteriol Orig 215:212–220. 4992024

[44] Smith GE, Hinde F, Westmoreland D, Berry PR, Gilbert RJ 1989 Infantile botulism. Arch Dis Child 64:871–872. doi:10.1136/adc.64.6.871. 2673055 PMC1792581

[45] Lin G, Tepp WH, Bradshaw M, Fredrick CM, Johnson EA 2015 Immunoprecipitation of native botulinum neurotoxin complexes from *Clostridium botulinum* subtype A strains. Appl Environ Microbiol 81:481–491. doi:10.1128/AEM.02817-14. 25362065 PMC4277567

[46] Kalb SR, Baudys J, Webb RP, Wright P, Smith TJ, Smith LA, Fernández R, Raphael BH, Maslanka SE, Pirkle JL, Barr JR 2012 Discovery of a novel enzymatic cleavage site for botulinum neurotoxin F5. FEBS Lett 586:109–115. doi:10.1016/j.febslet.2011.11.033. 22172278 PMC3263758

[47] Kalb SR, Lou J, Garcia-Rodriguez C, Geren IN, Smith TJ, Moura H, Marks JD, Smith LA, Pirkle JL, Barr JR 2009 Extraction and inhibition of enzymatic activity of botulinum neurotoxins/A1, /A2, and /A3 by a panel of monoclonal anti-BoNT/A antibodies. PLoS One 4:e5355. doi:10.1371/journal.pone.0005355. 19399171 PMC2670495

[48] Smith TJ, Lou J, Geren IN, Forsyth CM, Tsai R, Laporte SL, Tepp WH, Bradshaw M, Johnson EA, Smith LA, Marks JD 2005 Sequence variation within botulinum neurotoxin serotypes impacts antibody binding and neutralization. Infect Immun 73:5450–5457. doi:10.1128/IAI.73.9.5450-5457.2005. 16113261 PMC1231122

[49] Goodnough MC, Hammer B, Sugiyama H, Johnson EA 1993 Colony immunoblot assay of botulinal toxin. Appl Environ Microbiol 59:2339–2342. 8357267 10.1128/aem.59.7.2339-2342.1993 PMC182282

[50] Sambrook J, MacCallum P, Russell D 2001 Molecular cloning: a laboratory manual, 3rd ed. CSHL Press, New York, NY.

[51] Hall YH, Chaddock JA, Moulsdale HJ, Kirby ER, Alexander FC, Marks JD, Foster KA 2004 Novel application of an *in vitro* technique to the detection and quantification of botulinum neurotoxin antibodies. J Immunol Methods 288:55–60. doi:10.1016/j.jim.2004.02.011. 15183085

[52] Pellett S, Tepp WH, Clancy CM, Borodic GE, Johnson EA 2007 A neuronal cell-based botulinum neurotoxin assay for highly sensitive and specific detection of neutralizing serum antibodies. FEBS Lett 581:4803–4808. doi:10.1016/j.febslet.2007.08.078. 17889852 PMC2748649

[53] Schantz EJ, Kauter DA 1978 Standardized assay for *Clostridium botulinum* toxins. J Assoc Off Anal Chem 61:96–99.

[54] Hatheway CL 1988 Botulism, p 111–133. In Balows A, Hausler WH, Ohashi M, Turano MA (ed), Laboratory diagnosis of infectious diseases: principles and practice, vol 1 Springer-Verlag, New York, NY.

[55] Reed LJ, Muench H 1938 A simple method of estimating fifty percent endpoints. Am J Hyg 27:493–497.

[56] Maslanka SE, Sharma S, Johnson EA 2013 *Clostridium botulinum* and its toxins. In Tortorello ML, Doores S, Ito K, Salfinger Y (ed), Compendium of methods for the microbiological examination of foods. American Public Health Association, Washington, DC.

[57] Pellett S, Tepp WH, Toth SI, Johnson EA 2010 Comparison of the primary rat spinal cord cell (RSC) assay and the mouse bioassay for botulinum neurotoxin type A potency determination. J Pharmacol Toxicol Methods 61:304–310. doi:10.1016/j.vascn.2010.01.003. 20100585

