# Supplementary material for: Purification and Characterization of Botulinum Neurotoxin FA from a Genetically Modified Clostridium botulinum Strain
Source: mSphere. 2016 Feb 24;1(1):e00100-15. doi: 10.1128/mSphere.00100-15 (PMC4863619; doi:10.1128/mSphere.00100-15)
Supplement: Table S1 [file sph001162030st1.docx]

Table 1. Oligonucleotide primers used in this study.

Oligonucleotide primer Sequence (5’ – 3’)

Intron targeting

381|382s-IBS AAAAAAGCTTATAATTATCCTTAGTTCCCCTCGAAGTGCGCCCAGATAGGGTG

381|382s-EBS1d CAGATTGTACAAATGTGGTGATAACAGATAAGTCCTCGAAGATAACTTACCTTTCTTTGT

381|382s-EBS2 TGAACGCAAGTTTCTAATTTCGATTGGAACTCGATAGAGGAAAGTGTCT

EBS Universal CGAAATTAGAAACTTGCGTTCAGTAAAC

Intron sequencing

pMTL007-R1 AGGGTATCCCCAGTTAGTGTTAAGTCTTGG

Screening of clones by PCR and sequencing

B268F CAAACAATGATCAAGTTATTTAATAG

B506R TCATTTAAAACTGGCCCAGG

Generation of hybridization probe for the Erm gene

ErmF ATGAACAAAAATATAAAATATTCTCAAAAC

ErmR TTATTTCCTCCCGTTAAATAATAGATAACG
